# Intramedullary nailing versus external fixation in Gustilo type III open tibial shaft fractures: a meta-analysis of randomised controlled trials

**DOI:** 10.1007/s11751-016-0245-7

**Published:** 2016-02-26

**Authors:** Francesca Giovannini, Luigi de Palma, Andrea Panfighi, Mario Marinelli

**Affiliations:** Clinica di Ortopedia, Azienda Ospedaliero-Universitaria Ospedali Riuniti di Ancona, Università Politecnica delle Marche, Via Conca, Torrette, 60100 Ancona, Italy

**Keywords:** External fixation, Intramedullary nailing, Open tibia fractures

## Abstract

Open tibial shaft fractures are the most common of long-bone open fractures. Management of the fracture is either by intramedullary nailing (IMN) or by external fixation (EF). Since the literature does not indicate clearly which is more effective, a meta-analysis was conducted to establish which approach is more suitable to treat Gustilo type III fractures. MEDLINE, the Cochrane Central Register of Controlled Trials, EMBASE and CINAHL databases were searched for randomised controlled trials (RCT) describing IMN and EF treatment of Gustilo type III fractures. As of 15 November 2012, five RCT involving 239 patients had been published; the outcomes examined in this study are their surgical complications. Data analysis led complications to be grouped into infection, fracture healing problems (non-union, malunion) and “other complications” (vascular injury, revision surgery, soft tissue damage, mechanical failure and tibial malalignment). IMN was associated with lower rates of infection and fracture healing problems; the differences between the two approaches for “other complications” were not significant. The data indicate that IMN is the treatment of choice for Gustilo type III fractures.

## Introduction

The anteromedial aspect of the tibia is covered by a thin cutaneous layer, and as a result severe soft tissue injury with bone trauma, including fracture, is frequent at this site [1]. Lower limb fractures are closed in 77 % of patients and open in the remaining cases. Open fractures require emergency treatment that involves debridement, repair of soft tissue injuries (muscles, tendons), fracture reduction and stabilisation with external or internal fixation. Antibiotic therapy is required prior to surgery [2–5]. There is no consensus in the management of open fractures. The Gustilo–Anderson classification provides a guide to treatment. The classification is based on the extent of the skin wound exposing the fracture and of additional damage; fractures are divided into three types with the third type further divided into three subtypes [6, 7]. Open fractures of the tibial diaphysis are managed by four main approaches: (1) non-operative treatment, which includes a full-length plaster cast, a hinged brace allowing knee movement or functional braces that allow knee and foot movement; the other approaches involve surgical stabilisation which include: (2) plates; (3) intramedullary nailing (IMN); or (4) external fixation (EF). The use of plates was widespread in the 1960s and 1970s and is still popular in some parts of the world. Currently, the most widely used methods are IMN and EF [1].

## Aim of the study

The purpose of a meta-analysis is to examine issues on which published data are conflicting, to identify the best therapeutic approach based on key outcome measures. Despite being an extensively explored topic, data on the relative value of IMN and EF in managing Gustilo type III open tibial shaft fractures are conflicting. The aim of this meta-analysis is to establish the relative effectiveness of IMN and EF in treating these fractures.

## Materials and methods

The MEDLINE, Cochrane, Central Register of Controlled Trials, EMBASE and CINAHL databases were searched for randomised controlled trials (RCT) using “intramedullary nailing”, “external fixation”, “open tibia fracture” as the keywords. The search was conducted on 15 November 2012 and yielded 16 papers.

Paper selection was performed separately by two of the authors based on the following inclusion criteria: men and women aged 18 years or older; Gustilo type III open tibial shaft fracture; treatment with IMN or EF performed within 6 h of trauma; and assessment of surgical complications.

Five RCT comparing the two methods were included in the meta-analysis [8–12]. They involved a total of 239 patients who underwent surgical debridement, soft tissue repair and fracture reduction and fixation with IMN (irrespective of boring) or EF within 6 h. Complications were grouped into infection, fracture healing problems (malunion, non-union) and “other complications”. The latter group encompassed vascular injury, revision surgery, soft tissue damage, mechanical failure and tibial malalignment which were not addressed in all 5 RCT.

Data were tested with a Mantel–Haenszel (M–H) approach or fixed effects model. This required compiling a 2 × 2 contingency table (not shown) for each trial and outcome measure investigated (15 tables overall) and executing the three M–H steps: a test of homogeneity, an estimate of the strength of the association and calculation of the overall odds ratio (OR). Review Manager 5.2 software was used for generating the forest plot. This paper conforms to Preferred Reporting Items for Systematic Reviews and Meta-Analyses (PRISMA) guidelines.

## Results

Among the five RCT meeting the inclusion criteria [8–12], the study by Holbrook and co-workers did not describe the fracture subtypes considered; Tornetta et al. treated only Gustilo type IIIB fractures; Tu et al. and Mohseni et al. treated both IIIA and IIIB fractures; and Inan et al. considered only IIIA fractures. No study seemed to include IIIC fractures. We divided patients into two approach-based groups: IMN and EF. Of the 57 patients described by Holbrook et al. [8], 29 were treated by IMN and 28 by EF; 2 of the former patients had infection, 7 had fracture healing problems and 12 had “other complications”; of their EF patients 11 had infection, 9 had fracture healing problems and 11 had “other complications”. Tornetta et al. [9] included 29 patients, 14 with IMN and 15 with EF; of those treated with IMN 3 had infection, none had fracture healing problems and 5 had “other complications”; among those managed by EF, there were 6 cases of infection, 2 fracture healing problems and 8 “other complications”. Tu et al. [10] described 36 patients, 18 treated with IMN and 18 with EF; those receiving IMN had 4 infections, 3 fracture healing problems and 2 “other complications”, whereas patients treated with EF had 2 infections, 8 fracture healing problems and 3 “other complications”. Inan et al. [11] treated 61 patients, 29 with IMN and 32 with EF; of the IMN patients, 3 had infection, 4 had fracture healing problems and 7 had “other complications”; of the EF patients, 2 had infection, 4 had fracture healing problems and 3 had “other complications”. Mohseni et al. [12] considered 50 patients, 25 treated by IMN and 25 by EF; of those managed by IMN, 4 developed infection, 1 had fracture healing problems and 2 had “other complications”, whereas of those receiving EF 8 had infection, 8 had fracture healing problems and 3 had “other complications”.

The data from the 2 × 2 tables were used to obtain the forest plot for each outcome measure considered. The plot for the incidence of infection (Table 1) favours IMN due to a lower incidence of infection (OR = 0.48); the same applies to fracture healing problems (Table 2) (OR = 0.41). The results for “other complications” (Table 3) are not significant (OR = 1.14), providing no clear indication.

**Table 1:**
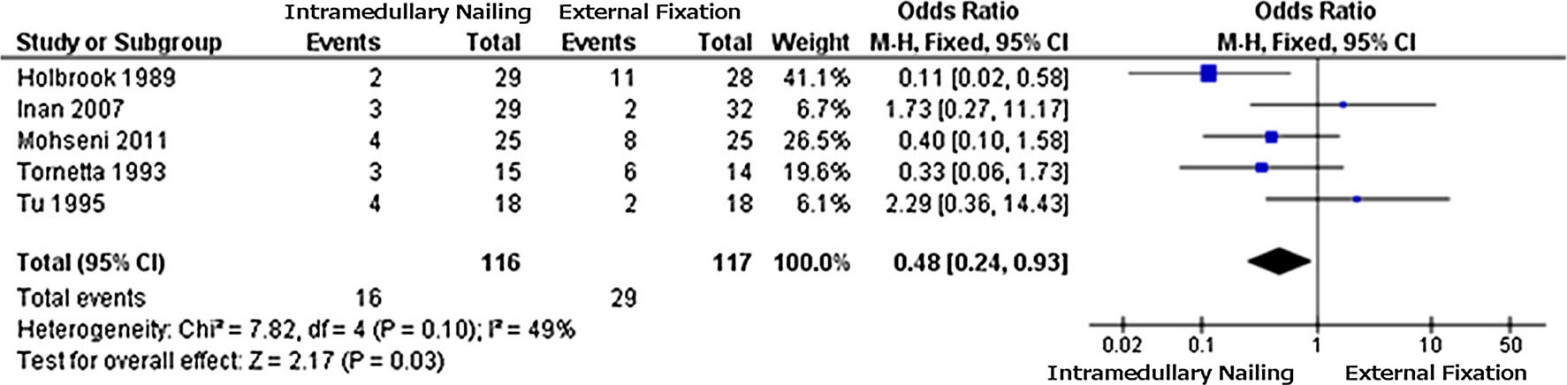
Forest plot 1: infections

*Black boxes* indicate the odds ratio (OR) of each study; the *line* issuing from each box is the 95 % confidence interval (CI) for that study. *Box* size is related to the weight attributed to each study in the meta-analysis. The *black diamond* represents the combined OR for all studies, and its width corresponds to 95 % CI bounds. The *vertical line* is the line of no effect

**Table 2:**
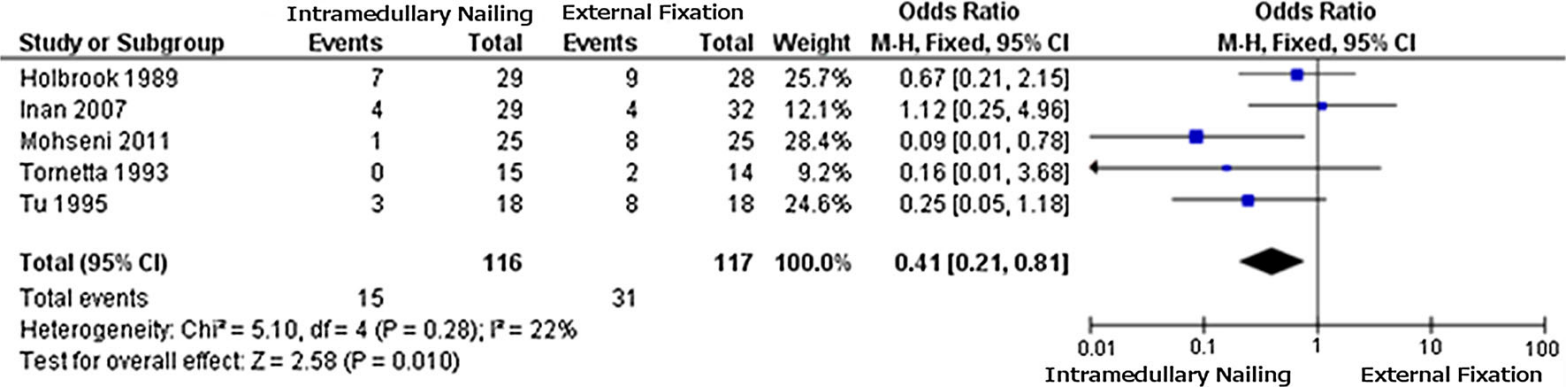
Forest plot 2: fracture healing problems

Odds ratios and confidence intervals as in Table 1

**Table 3:**
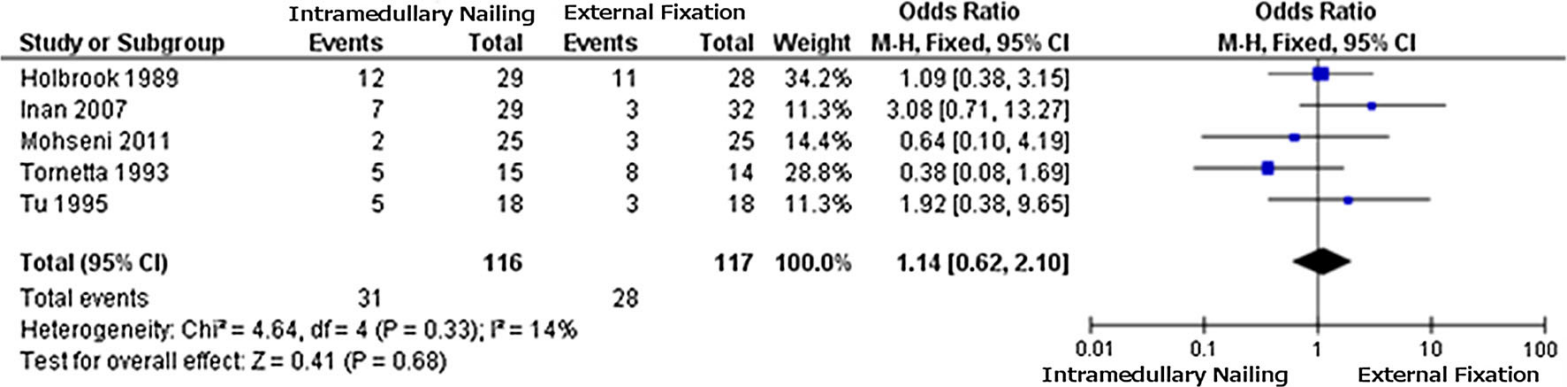
Forest plot 3: other complications

Odds ratios and confidence intervals as in Table 1

## Discussion

Several clinical trials have compared fracture management with IMN and EF; the two approaches are also applied to treat open tibial shaft fractures. Treatment selection is a function both of patient presentation and of the surgeon’s experience. EF involves shorter operating times and is more suitable in polytrauma patients; however, it is not well tolerated and carries a higher incidence of complications including non-union, delayed union and re-fracture. The advantages of IMN are shorter healing time, earlier load-bearing (albeit initially partial), earlier ambulation and a lower rate of complications (even though some studies report a higher infection rate [13–15]). Given the longer operating time, IMN tends to be used in patients with isolated fractures. All IMN procedures reported in the five RCT were primary procedures.

This meta-analysis compares the relative benefits of the two main approaches to primary surgical treatment of Gustilo type III fractures: IMN and EF. The data from each trial were grouped in relation to three main outcome measures: infection, fracture healing problems and “other complications”. The latter group included a number of major complications that were not assessed individually in all five RCT: vascular injury, revision surgery, soft tissue damage, mechanical failure and tibial malalignment. The data from each trial were entered into contingency tables, one table per trial and per outcome measure (*n* = 15), and tested with the M–H approach, to establish the better therapeutic approach.

The conclusions that can be drawn from the meta-analysis are clearly subject to the limitations of the original studies. Fracture classification data (type IIIA, IIIB and IIIC) were not consistently specified: in particular, Holbrook et al. did not describe the fracture subtype of their patients; Tornetta et al. treated only type IIIB; Tu et al. and Mohseni et al. failed to distinguish IIIA from IIIB fractures; and Inan managed only IIIA injuries. There were probably no type IIIC fractures. The results were processed using forest plots, which provide a graphic representation of the results of each study, the point estimates and the overall estimate, which are very effective in the first interpretation of meta-analysis data. The question whether Gustilo type III tibial fractures should be treated with non-reamed IMN or EF was addressed in a previous meta-analysis [16], where Fang et al. highlighted a lower malunion rate with non-reamed IMN and no significant differences in deep infection, non-union and time to union. They included prospective, randomised, case–control and cohort studies and examined deep infection, malunion, non-union and time to union. Their meta-analysis did not highlight clear advantages for either approach except in relation to the malunion rate, which, however, is not a key factor determining treatment selection. We included only RCT describing IMN and EF and assessing infections, fracture healing problems and “other complications”.

## Conclusions

The results of our meta-analysis show that IMN is the more effective approach to Gustilo type III open tibial fractures, because of the lower incidence of infectious events and fracture healing problems. The forest plots show this clearly. As regards the “other complications”, there are no significant differences between the techniques. These findings are not conclusive. Although the present meta-analysis shows IMN as the better option, each department should analyse their outcomes to see whether their data are in line with these findings.
